# Irreversible alteration of extracellular vesicle and cell-free messenger RNA profiles in human plasma associated with blood processing and storage

**DOI:** 10.1038/s41598-022-06088-9

**Published:** 2022-02-08

**Authors:** Hyun Ji Kim, Matthew J. Rames, Samuel Tassi Yunga, Randall Armstrong, Mayu Morita, Anh T. P. Ngo, Owen J. T. McCarty, Fehmi Civitci, Terry K. Morgan, Thuy T. M. Ngo

**Affiliations:** 1 Cancer Early Detection Advanced Research Center, Knight Cancer Institute (CEDAR), Oregon Health and Science University, 2720 SW Moody Ave, KR-CEDR, Portland, OR 97201 USA; 2 Department of Biomedical Engineering, Oregon Health and Science University, Portland, OR USA; 3 Department of Molecular and Medical Genetics, Oregon Health and Science University, Portland, OR USA; 4 Department of Pathology, Oregon Health and Science University, Portland, OR USA

**Keywords:** Biomarkers, Biological techniques

## Abstract

The discovery and utility of clinically relevant circulating biomarkers depend on standardized methods that minimize preanalytical errors. Despite growing interest in studying extracellular vesicles (EVs) and cell-free messenger RNA (cf-mRNA) as potential biomarkers, how blood processing and freeze/thaw impacts the profiles of these analytes in plasma was not thoroughly understood. We utilized flow cytometric analysis to examine the effect of differential centrifugation and a freeze/thaw cycle on EV profiles. Utilizing flow cytometry postacquisition analysis software (FCMpass) to calibrate light scattering and fluorescence, we revealed how differential centrifugation and post-freeze/thaw processing removes and retains EV subpopulations. Additionally, cf-mRNA levels measured by RT-qPCR profiles from a panel of housekeeping, platelet, and tissue-specific genes were preferentially affected by differential centrifugation and post-freeze/thaw processing. Critically, freezing plasma containing residual platelets yielded irreversible ex vivo generation of EV subpopulations and cf-mRNA transcripts, which were not removable by additional processing after freeze/thaw. Our findings suggest the importance of minimizing confounding variation attributed to plasma processing and platelet contamination.

## Introduction

Circulating extracellular vesicles (EVs) and cell-free RNA (cfRNA) are promising biomarkers for early cancer detection^1^. EVs are a heterogeneous mixture of vesicles of varying size and composition that are released from cells^2–5^. Since EVs are either derived from the plasma membrane or the involvement of multivesicular endosome fusion with the cell surface, they have cell-specific antigens on their surface that may be antibody labelled for imaging and/or isolation in –omics analyses^6–8^. There is increasing evidence that EVs may transport a variety of proteins and nucleic acids, including being a potential carrier of cfRNA^9,10^. Cell-free messenger RNAs (cf-mRNA) specifically are protein coding mRNA molecules in plasma that may serve as biomarkers^11,12^. Since EVs may transport diverse extracellular RNAs, including cf-mRNA, there is an intense interest in the combination of these analytes for blood-based cancer diagnosis^13,14^.

Previous studies have suggested that ex vivo platelet activation and fragmentation affect EV profiles in serum and plasma^2,15–21^. Thus, the International Society of Extracellular Vesicles (ISEV) and International Society on Thrombosis and Haemostasis (ISTH) have recommended general platelet-poor plasma processing conditions for EV analysis^22,23^. However, how specific preanalytical variables can influence EV subpopulations was not thoroughly characterized. Others have shown that residual platelets also significantly affect plasma microRNA levels solely due to differences in blood processing methods^16,17^. However, no prior studies have specifically examined changes in cf-mRNA. Since common blood processing conditions for biobanking may not produce platelet-poor plasma^16^, as guided by ISEV and ISTH, additional processing on banked samples after thawing may mitigate the effect of platelet activation on EVs and cf-mRNA analysis. However, which subpopulations of ex vivo generated EVs and cf-mRNA subtypes are removable or retained is unknown.

Despite the growing body of literature describing the impact of blood processing on EVs, standardization through light scatter calibration was not widely adopted in these studies to analyze EV subpopulations using flow cytometry. Flow cytometry has been increasingly utilized to characterize the heterogeneity of EV surface markers^2,16,24–29^. Nonetheless, standardizing nanoscale flow cytometry for sub-micron sized EV detection can be challenging due to varying instrument settings and resolution^26–28^. Recent efforts to ameliorate this have focused on Mie scattering theory modeling^30–34^. An estimated relative size of an EV population can be derived from a given scatter intensity provided an assumed refractive index and specific optical configuration in a flow cytometer^32–34^. Although quantifying the exact refractive index of EVs is challenging, previous measurements by either nanoparticle tracking analysis or fluorescence lifetime imaging microscopy suggested a potential range from 1.37 to 1.45^35–37^. Using National Institutes of Standards and Technology (NIST) traceable bead standards with known diameters and refractive indices, scatter-diameter curves can be generated via postacqusition analysis software^30–34^. Given an effective refractive index of EVs, established scatter-diameter curves yielded reproducible EV measurement between instruments^30–34^.

In this study, we systematically examined the variation of both EV and cf-mRNA subpopulations in human plasma due to blood processing and freeze thaw effect after − 80 °C storage. EVs were analyzed by flow cytometry with standardized size and fluorescent calibration, and cf-mRNA levels were measured by multiplex RT-qPCR. We compared plasma derived from single spin (S1: 1000 × *g* centrifugation for 10 min) and double spin (S2: 15,000 × *g* secondary spin for 10 min after the initial single spin S1) analyzed freshly and after freezing. We examined how post-freeze/thaw processing removes and retains specific EV subpopulations as well as cf-mRNA originated from platelets, common cell types and tissue specific cells. Our analysis revealed subpopulations of EVs and cf-mRNA were irreversibly altered ex vivo in association with blood processing and freeze/thaw effects after storage.

## Materials and methods

### Blood sample collection and processing

All experimental protocols were reviewed and approved by the Oregon Health & Science University Institutional Review Board. All methods were carried out in accordance with relevant guidelines and regulations. Blood samples from healthy individuals were obtained from the Cancer Early Detection Advanced Research center (CEDAR) at Oregon Health and Science University. All samples were collected under institutional review board (IRB) approved protocols with informed consent from all participants for research use. Whole blood was collected from healthy individuals with 8.5 ml in ACD-A (BD Vacutainer, Becton Dickinson, cat. 364606), 10 ml in K2EDTA tubes (BD Vacutainer, Becton Dickinson, cat. 36643), 10 ml in heparin (BD Vacutainer, Becton Dickinson, cat. 367874), or 3 ml in sodium citrate tubes (BD Vacutainer, Becton Dickinson, cat. 369714) via antecubital vein puncture using a 21G butterfly needle (BD Vacutainer, Becton Dickinson, cat. 367281). Tubes were transported vertically at room temperature before processing. Within 1 h of blood withdrawal, 10 ml of whole blood was centrifuged at 1000 × *g* for 10 min at room temperature with the highest acceleration and deceleration setting at ‘9’ using Eppendorf 5810-R centrifuge with S-4-104 Rotor. Plasma was collected until 10 mm above the buffy coat and was labelled as S1. To obtain double spun plasma, S1 plasma was centrifuged in Eppendorf 5424R centrifuge at 15,000 × *g* for 10 min at room temperature. The resulting supernatant of platelet-depleted plasma was collected and labelled as S2. S1 and S2 plasma samples did not undergo a freeze/thaw cycle. Plasma samples that were frozen at − 80 °C and thawed at room temperature were labelled as S1FR and S2FR respectively. For post-thaw processing, S1FR was centrifuged in Eppendorf 5424R centrifuge at 15,000 × *g* for 10 min at room temperature. The resulting supernatant was carefully transferred and designated as S1FRS2. All samples were collected and processed using a uniform protocol at Oregon Health and Science University. The overall schematic diagram of plasma processing steps was described in Supplementary Fig. S1.

### Platelet counting using improved Neubauer haemocytometer

The platelet count was measured by the improved Neubauer haemocytometer (VWR Scientific Products, Piscataway, NJ) by two independent, experienced researchers. The total number of platelets were counted from the central 1 × 1 mm area consisting of 25 groups of 16 squares separated by closely ruled triple lines.

### Flow cytometry set-up for light scatter and fluorescence calibration

Beckton-Dickinson FACSAria Fusion equipped with 488 nm (60 mW), 561 nm (100 mW), and 640 nm (100 mW) lasers was used. For optimal configuration of submicron size detection, 0.1 µm size filter was applied to the sheath fluidic system to reduce sheath fluid noise. The sample flow rate was set at 1, which was measured by mass discharge^30^ and determined to be 45 μl/min. Timed collections were recorded for 60 s. Data collection was set using the SSC trigger threshold value of 200 using scatter wavelength at 488 nm. In order to calibrate light scattering, 152, 203, 303, 401, 510, and 600 nm polystyrene NIST-traceable beads (ThermoFisher Scientific, cat. 3150A, 3200A, 3300A, 3400A, 3500A, and 3600A) were serially diluted in 0.1 μm filtered D-PBS without calcium and magnesium (ThermoFisher Scientific, cat. 14190250). A minimum of 5000 events were recorded for 60 s. Particle diameter and scatter relationship was established utilizing FCMpass software (v3.09, http://nanopass.ccr.cancer.gov)^32–34^. Median SSC-H intensity in arbitrary units was converted to standardized unit in EV diameter. To approximate EV diameter size, the average of effective refractive index (RI) data based upon published measurements were used. Detailed instructions for light scattering calibration based on a core–shell structure to model EVs were followed (Shell RI = 1.4800, Core RI: 1.3800, and shell thickness: 5 nm)^32^. For fluorescence calibration, Quantum Alexa Fluor 647 Molecule Equivalent Soluble Fluorochrome (MESF) (Bangs Laboratories, cat. 647), Quantum Alexa Fluor 488 MESF (Bangs Laboratories, cat. 488), or Quantum PE MESF (Bangs Laboratories, cat. 827) were used. Data collection was set using the FSC trigger threshold value of 5000 and analyzed using FSC-A vs SSC-A in arbitrary units. Utilizing FCMpass software, the fluorescent intensity in arbitrary units were converted to MESF standardized units. All measurements were analyzed using FlowJo software.

### Fluorescent antibody labeling of differentially processed plasma for flow cytometry

To fluorescently label EV surface proteins, 5 µl of plasma was incubated with 5 µl of antibody mix prepared after established dilution series. CD9 Alexa Fluor 647 (R&D system, clone: #209306, cat. FAB1880R-100 μg) was diluted to a final concentration of 0.001 mg/ml for staining. CD63 Alexa Fluor 488 (Thermofisher scientific, clone: MEM-259, cat. MA5-18149, concentration 0.26 mg/ml) was diluted to a final concentration of 0.0013 mg/ml for staining. CD41 PE (Biolegend, clone: HIP8, cat. 303706) was diluted to a final concentration of 0.001 mg/ml for staining. For isotype controls, mouse IgG2B Alexa Fluor 647 conjugated isotype control (R&D system, cat. IC0041R), mouse IgG1 Alexa Fluor 488 conjugated isotype control (Thermofisher Scientific, cat. MA518167), and mouse IgG1, k PE conjugated isotype control (Biolegend, cat. 981804) were used at the same concentration as matched stained controls and were recorded at the same dilution as stained and unstained samples. Incubation was done for 3 h at room temperature in the dark. The stained EV samples were further diluted 200-fold with 0.1 μm filtered D-PBS without calcium and magnesium prior to acquiring the data using an abort rate of < 5% and keeping the threshold rate below 20,000 events per second. To account for the electronic abort rate due to nanoparticle coincidence (also known as “swarming”), stained samples were serially diluted and validated via consistent median fluorescent intensity across plasma dilutions. A buffer-only control of 0.1 µm-filtered DPBS without calcium and magnesium was recorded at the same flow cytometer acquisition settings as all other samples, including triggering threshold, voltages, and flow rate. The buffer-only control had a count of < 1000 events per second.

### Characterization of platelets using flow cytometry

For counting platelets in differentially processed plasma, blood samples from three healthy individuals were obtained in 10 ml K2EDTA tubes (BD Vacutainer, Becton Dickinson, catalog number: 36643). Plasma was processed using single spin at 1000 × *g* (S1). To obtain double spun plasma, S1 plasma was centrifuged in Eppendorf 5424R centrifuge at 15,000 × *g* for 10 min at room temperature (S2). To investigate the CD9, CD63, and CD41 expression, 5 µl of S1 and S2 plasma was incubated with 5 µl of antibody mix prepared after established dilution series. CD9 Alexa Fluor 647 (R&D system, clone: #209306, cat. FAB1880R-100 μg) was diluted to a final concentration of 0.001 mg/ml for staining. CD63 Alexa Fluor 488 (Thermofisher scientific, clone: MEM-259, cat. MA5-18149, concentration 0.26 mg/ml) was diluted to a final concentration of 0.0013 mg/ml for staining. CD41 PE (Biolegend, clone: HIP8, cat. 303706) was diluted to a final concentration of 0.001 mg/ml for staining. After 3 h of incubation at room temperature in the dark, 50 µl of CountBright beads (Thermo Fisher Scientific, cat. C36950), 2 µl of stained platelet samples, and 148 µl of 0.1 µm filtered D-PBS without calcium and magnesium (Thermo Fisher Scientific, cat. 14190250) were added prior to acquiring the data using Beckton-Dickinson FACSAria Fusion. Using FSC vs. SSC dot-plot, CountBright beads were distinguished and analyzed in the defined gates^38^. To calculate platelet amount, CD41^+^ platelet counts in CD41 vs SSC dot-plot were used^38^. The concentration of platelets (platelets per µl) was calculated using the following formula: A = ((B × C)/(D × E)) × F, where A is the concentration of platelets (platelets per µl), B is the gated events for platelet population, C is the volume of CountBright beads, D is the gated events for CountBright beads, E is the volume of platelet samples (µl), and F is the CountBright bead concentration (beads per µl) from the manufacturer.

### RT-qPCR profiling of cell free mRNA

For characterizing the effect of freeze thaw on cell free mRNA expressions, RNA was extracted using plasma processed with S1, S2, S1FR, S2FR, and S1FRS2 conditions. Cell free mRNA was isolated by using plasma/serum circulating and exosomal RNA purification Kit (Norgen Biotek, cat. 42800) followed by 10X Baseline-ZERO DNase treatment (Epicentre, cat. DB0715K). DNase treated RNA samples were purified and further concentrated using RNA clean and concentrator (Zymo Research, cat. R1014). The purified RNA samples were assayed by RT-qPCR using custom selected 16 primers targeting MTND2, PPBP, B2M, PF4, ACTB, CORO1C, GSE1, GAPDH, SMC4, HBG1, NUSAP1, MIKI67, FGB, APOE, FGG, and ALB. Template RNA was mixed with Superscript III One-step RT-PCR system with Platinum Taq DNA polymerase (Invitrogen, cat. 11-732-020) to generate cDNA according to the protocol. PCR amplification products were treated with Exonuclease I (New England Biolabs, cat. M0293L) to digest single stranded primers at 37 °C for 30 min followed by inactivation of enzymes at 80 °C for 15 min. For RT-qPCR, cDNA from preamplification was diluted 1:80 and set-up in 96-well plates with SsoFast EvaGreen supermix with low ROX (BioRad, cat. 1725211) with above primers at 10 µM. QuantStudio 7 Flex (Applied Biosystems) was used to run RT-qPCR assay according to manufacturer’s recommended cycling conditions.

### Statistical analysis

Significance for platelet count measurements using the improved Neubauer haemocytometer and flow cytometry across differential centrifugation were both assessed using the Wilcoxon test. To determine the impact of overall preanalytical factors, statistical analysis was performed on CD9^+^, CD63^+^, or CD41^+^ EVs on specific gated populations across differential centrifugation and freeze/thaw processing. The significance of individual preanalytical factor comparisons were determined using Tukey’s multiple comparison test. *P* values < 0.05 were considered statistically significant (*P < 0.05, **P < 0.01, ***P < 0.001, and ****P < 0.0001). Analyses were conducted using R package.

## Results

### Light scattering and fluorescence calibration

**Figure 1:**
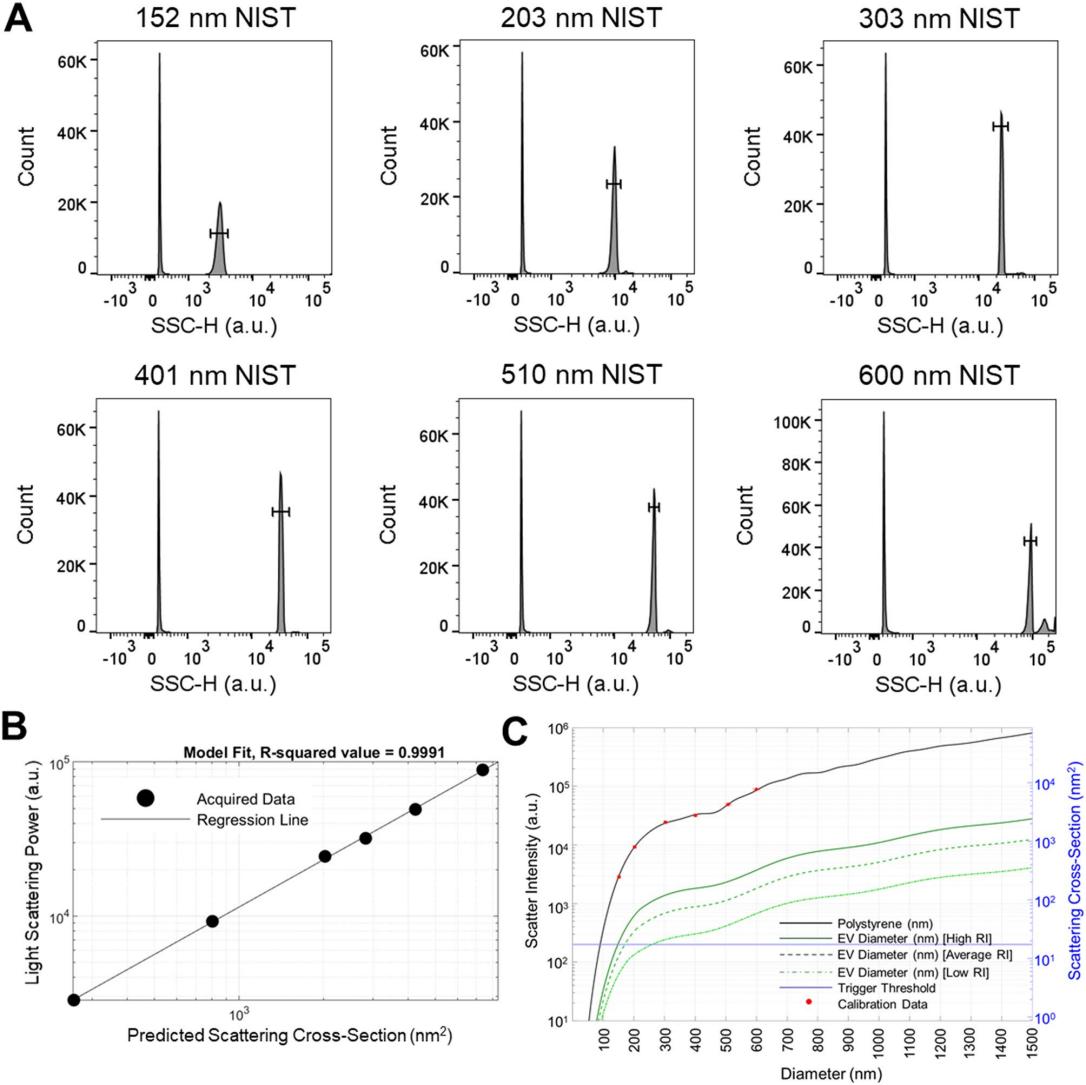
Light scattering calibration. (**A**) Histogram of NIST-traceable polystyrene beads (152, 203, 303, 401, 510, and 600 nm) are shown using side scattering (SSC-H) on a bioexponential scale using FlowJo. Each bead size relative to SSC-H is identified to obtain median side scattering in arbitrary units (a.u.) for light scattering calibration. (**B**) Regression plot of acquired light scattering power in arbitrary units compared to the predicted scattering cross-section in nm^2^ is calculated using FCMpass software. (**C**) Scatter-diameter curve showing light scatter intensity relationships with EV diameter established in FCMpass software. The acquired NIST-traceable polystyrene bead scattering intensity are overlaid with the predicted scattering data for NIST-traceable polystyrene beads with refractive index of 1.5900. The scatter-diameter relationship given high, average, and low effective EV refractive indices are shown, which can be used to estimate EV diameter from corresponding scattering intensity in arbitrary units.

**Figure 2:**
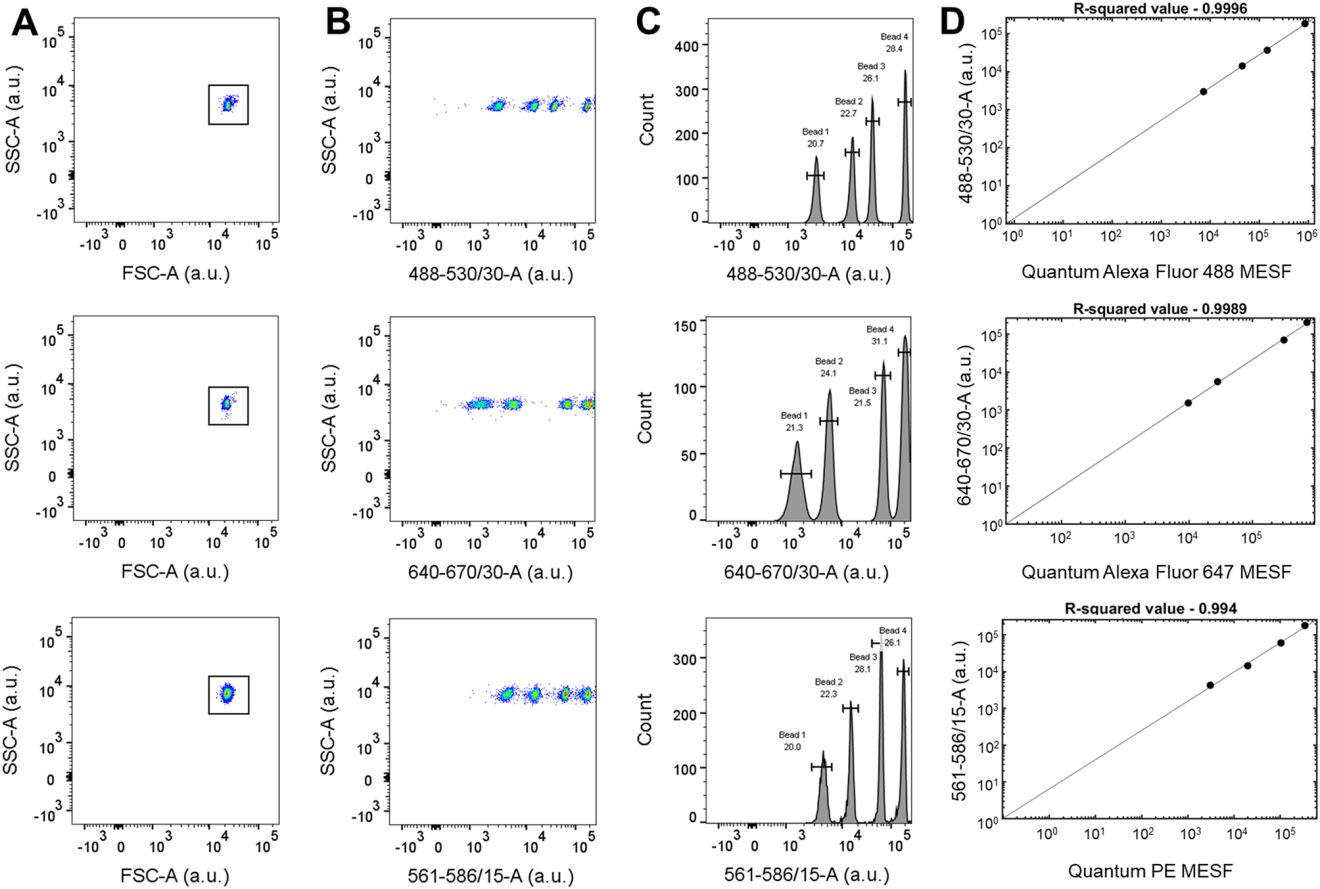
Fluorescence calibration. (**A**) Representative flow cytometry dot plots of Quantum Alexa Fluor 488 MESF (top), Quantum Alexa Fluor 647 MESF beads (middle), and Quantum PE MESF (bottom) gated using SSC-A and FSC-A in arbitrary units (a.u.) from FlowJo. (**B**) The gated beads are shown in each fluorescence channel (488–530/30-A, 640–670/30-A, and 561–586/15-A respectively) against SSC-A in arbitrary units using FlowJo. (**C**) Histogram of Quantum Alexa Fluor 488 MESF, Quantum Alexa Fluor 647 MESF, and Quantum PE MESF beads are shown using fluorescent intensity in arbitrary units from FlowJo. The subsets of each bead differing in fluorescence intensity are drawn to obtain median fluorescence in arbitrary units for fluorescence calibration. (**D**) Regression of acquired fluorescence intensity in arbitrary units to MESF bead reference values for each population established in FCMpass software.

We calibrated the flow cytometer Beckton-Dickinson FACSAria Fusion using National Institutes of Standards and Technology (NIST) traceable size standard beads (152, 203, 303, 401, 510, and 600 nm) and Quantum Molecules of Equivalent Soluble Fluorophore (MESF) to establish standardized units for light scatter and fluorescence respectively^32–34^. For light scatter, each bead sample was analyzed at the same acquisition setting until > 5000 bead events were recorded. The histogram of each sized bead population revealed distinct side scattering in arbitrary units, where a progressive increase in SSC-H with increasing NIST bead diameter (152–600 nm) was observed (Fig. 1A). The median light scatter statistic of each bead size gated population was inputted into flow cytometry postacquisition analysis software (FCMpass) to calibrate light scattering^32–34^. The collection half-angle of our system, which is important to quantify the amount of light reaching a detector in absolute units, was determined to be 45.3° using FCMpass software. Utilizing the side scattering collection angle, recorded side scattering value in arbitrary units was standardized to predicted scattering cross-section using Mie theory^32–34^. The linear regression between our observed light scattering power in arbitrary units and predicted scattering cross-section resulted in R-squared value = 0.9991 (Fig. 1B). The acquired scattering intensity of standard beads (red dots) was plotted on modelled data (black line) for polystyrene beads, which revealed the model fitted actual data accurately (Fig. 1C). After scatter-diameter relationship for EVs is extrapolated using FCMpass software, the measured scatter signal for polystyrene beads corresponding to vesicle diameter is revealed (Fig. 1C). Approximate diameters of EVs was calculated using the average of effective EV refractive index (Shell RI = 1.4800, Core RI: 1.38000, and shell thickness: 5 nm)^32–34^. Next, we performed fluorescence calibration using forward scatter as the trigger threshold to gate micron-sized MESF beads. MESF beads for each fluorophore was analyzed until > 5000 bead events were recorded. While FSC-A vs. SSC-A revealed a single microsphere population, four fluorescent microspheres were observed with varying fluorescent intensity in arbitrary units (Fig. 2A, B). The median fluorescence statistic of each bead fluorescence gated population was inputted into flow cytometry postacquisition analysis software (FCMpass) to calibrate fluorescence (Fig. 2C). The relationship between MESF bead reference values and acquired fluorescence in arbitrary units was established to calibrate fluorescence (Fig. 2D).

### Flow cytometry reveals distinct vesicle populations differentially affected by blood processing condition

**Figure 3:**
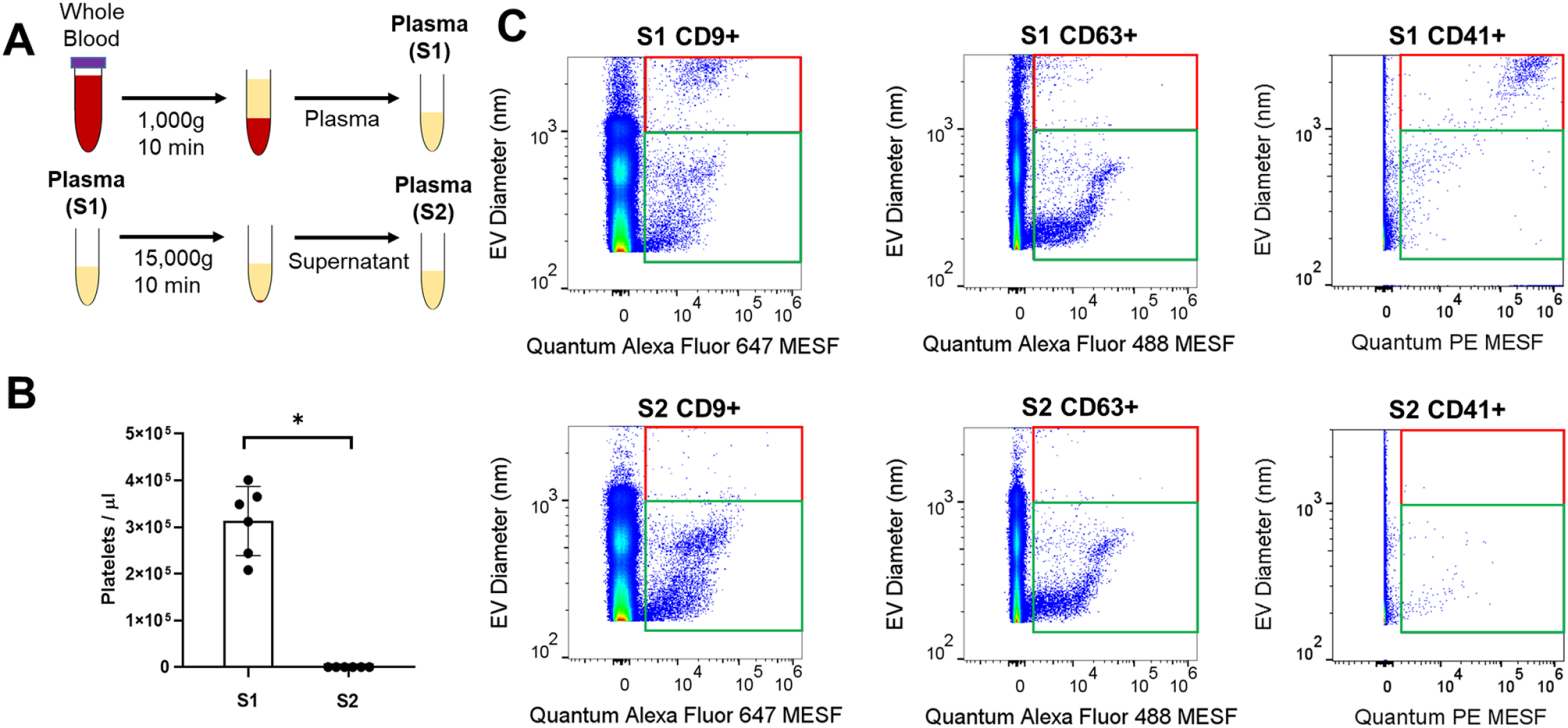
Effect of differential centrifugation on EVs using flow cytometry. (**A**) Schematic diagram of differentially processed plasma using single spin (S1: 1000 × *g* centrifugation) and double spin (S2: 15,000 × *g* secondary spin after the initial single spin S1). (**B**) Platelet concentration in differentially processed plasma from three healthy individuals (n = 3) was measured in independent technical replicates using a haemocytometer. The error bar represented standard deviations for the indicated blood processing conditions. *P*-value was calculated using Wilcoxon test (*P < 0.05). (**C**) Representative flow cytometry dot plot of EV diameter (nm) versus fluorescent intensity in Quantum Alexa Fluor MESF units for S1 and S2 using FlowJo. Quantum Alexa Fluor 647 MESF was used for Alexa Fluor 647 conjugated CD9 stained plasma, Quantum Alexa Fluor 488 MESF was used for Alexa Fluor 488 conjugated CD63 stained plasma, and Quantum PE MESF was used for PE conjugated CD41 stained plasma. Events were gated into two subpopulations: 150 to 1000 nm (green box) and from 1000 to 3000 nm (red box).

After establishing light scatter and fluorescence calibration, we investigated the impact of differential centrifugation on plasma EVs using the flow cytometer. Whole blood was collected in EDTA, and plasma was differentially processed using single centrifugation at 1000 × *g* for 10 min (S1) and double centrifugation (S2: 15,000 × *g* secondary spin for 10 min after the initial single spin S1) (Fig. 3A, Supplementary Fig. S1). Complete counts of residual platelets in plasma were measured using a haemocytometer and flow cytometry with platelet marker CD41. Using the haemocytometer, single spun plasma S1 contained an average platelet concentration of 313 ± 74 thousand/µl while secondary spin resulted in the removal of more than 99.99% of residual platelets in S2 (Fig. 3B). Using flow cytometer, single spun plasma S1 contained an average CD41^+^ platelet concentration of 127 ± 32 thousand/µl while secondary spin resulted in the removal of more than 99.90% of residual platelets in S2, consistent with the reduction measured by the haemocytometer (Supplementary Fig. S2A). For EV analysis, plasma was stained with anti-CD9, anti-CD63, and anti-CD41 fluorescent antibodies and measured by flow cytometry. The fluorescently positive gated data revealed that there are distinct populations in EV diameter distribution ranging between 150 and 3000 nm (Fig. 3C). It is noted that the subset of EVs within 150–1000 nm range at around 500 nm is an artifact of Mie scattering calibration from our calculated flow cytometer collection angle and geometry. Specifically, this corresponds to a plateau in the scatter-diameter curve from ~ 400–480 nm using predicted EV light scattering from the estimated average EV refractive index employed in our model (Fig. 1C). Welsh et al. reported a similar observation, suggesting that a plateau from the scatter-diameter curve resulted in an artifact between 400 and 480 nm accordingly^32^. Therefore, we gated EVs into two populations: 150–1000 nm which may be comprised of small and medium EVs, and 1000–3000 nm comprised of large EVs and platelets^30^. Notably, we observed much less 150–1000 nm CD41^+^ EVs for both S1 and S2 compared to 150–1000 nm CD9^+^ and CD63^+^ EVs. Flow cytometer assay controls included unstained samples, isotype controls, serial dilution of stained plasma, and antibody with buffer alone (Supplementary Fig. S3). Serial dilution of stained plasma showed the linear detection of EVs while the median fluorescence intensity remained constant, suggesting that EVs were detected and counted as single particles via flow cytometry. Plasma condition at S2 resulted in a clear reduction in 1000–3000 nm populations compared to S1 while the 150–1000 nm EVs remained similar for plasma from both processing conditions (Fig. 3C).

### Freezing of platelet containing single spun plasma generates ex vivo EVs

**Figure 4:**
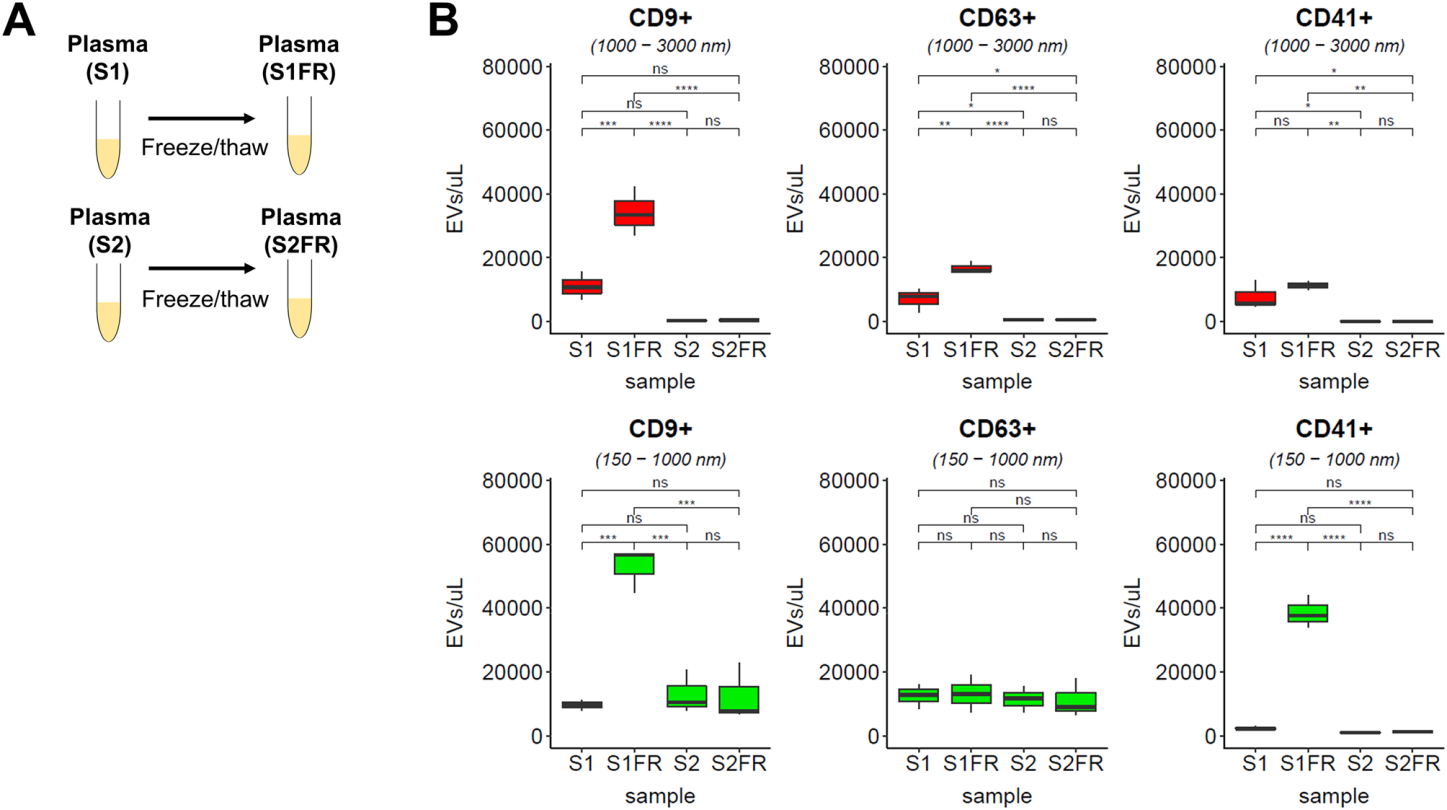
Effect of a freeze thaw cycle on EVs using flow cytometry. (**A**) Schematic diagram of differentially processed plasma (S1, S2) and respective freeze thaw processes (S1FR, S2FR). (**B**) Box plot of CD9^+^, CD63^+^, and CD41^+^ of gated events from 1000–3000 nm (red) and 150–1000 nm (green) for S1, S1FR, S2, and S2FR using R. CD9^+^, CD63^+^, CD41^+^ events were converted to concentrations using calibrated flow rate in a given acquisition time. EV concentration defined as the number of EVs per μl was determined by number of EVs detected in a given sample volume multiplied by the dilution factor. The sample volume was calculated by the product of measured flow rate and acquisition time. Statistical significance were obtained from three healthy volunteers for each freeze thaw processing condition using Tukey’s multiple comparisons (ns = not significant, *P* > 0.05; *P < 0.05, ***P < 0.001, ****P < 0.0001).

Previous studies have suggested that ex vivo platelet activation and fragmentation generate EVs^2,23,39,40^. We noted that single spun plasma S1 contained a high level of residual platelets (Fig. 3B). We first tested CD9, CD63, and CD41 expression on platelets and observed platelets expressed CD63, CD9, and CD41 (Supplementary Fig. S2B and C). Therefore, we examined how the freeze/thaw effect on plasma containing residual platelets impacts EV profiles using anti-CD9, anti-CD63, and anti-CD41 fluorescent antibodies. We compared EVs measured freshly (S1 and S2) with EVs after a freeze/thaw cycle (S1FR and S2FR) (Fig. 4A, Supplementary Fig. S4). The 1000–3000 nm CD9^+^ EV concentration in S1 plasma sample was around 11,103/µl while the 150–1000 nm CD9^+^ EV concentration in S1 plasma sample was around 9610/µl (Fig. 4B). We observed remarkably increased CD9^+^ EVs for both 1000–3000 nm and 150–1000 nm populations after single freeze/thaw cycle of S1 plasma (S1FR vs. S1, *P* < 0.001). On average, we observed a threefold increase for 1000–3000 nm CD9^+^ EVs and a 5.5-fold increase for 150–1000 nm CD9^+^ EVs between S1FR and S1. The 1000–3000 nm CD9^+^ EV concentration in S2 plasma sample was around 381/µl while the 150–1000 nm CD9^+^ EV concentration in S2 plasma sample was more comparable to S1 at around 12,976/µl (Fig. 4B). However, we observed no significant changes in CD9^+^ EVs occurred after single freeze/thaw cycle of S2 plasma samples for either size (S2FR vs. S2, ns). Similarly, the 1000–3000 nm CD63^+^ EVs were significantly increased in single spun plasma after freeze/thaw (S1FR vs. S1, *P* < 0.01) while remaining the same for double spun plasma (S2FR vs. S2, ns) (Fig. 4B). The concentration of CD63^+^ EVs in S1 plasma was around 6997/µl and 12,493/µl for 1000–3000 nm and 150–1000 nm respectively, while the concentration of CD63^+^ EVs in S2 plasma was around 532/µl and 11,486/µl for 1000–3000 nm and 150–1000 nm respectively. On average, we observed a 2.4-fold increase for 1000–3000 nm CD63^+^ EVs between S1FR and S1. In contrast, the 150–1000 nm CD63^+^ EVs were statistically unchanged with respect to either spin freeze/thaw cycle (Fig. 4B). When looking at the changes for CD41^+^ EVs, the 150–1000 nm populations increased remarkably after a single freeze/thaw cycle of S1 plasma (S1FR vs. S1, *P* < 0.0001) (Fig. 4B). The 1000–3000 nm CD41^+^ EV concentration in S1 plasma sample was around 7847/µl while the 150–1000 nm CD41^+^ EV concentration in S1 plasma sample was around 2365/µl. For single spun plasma, while 1000–3000 nm CD41^+^ EVs showed no significant change (S1FR vs. S1, ns), we observed a 16.3-fold increase for 150–1000 nm CD41^+^ EVs between S1FR and S1. The 1000–3000 nm CD41^+^ EV concentration in S2 plasma sample was around 77/µl while the 150–1000 nm CD41^+^ EV concentration in S2 plasma sample was around 933/µl (Fig. 4B). Overall, a single freeze/thaw cycle on double spun plasma resulted in no significant change for CD9^+^, CD63^+^, and CD41^+^ EVs for either size ranges (S2FR vs S2, ns). To confirm the nature of EVs which are sensitive to detergent lysis, we applied detergent to disrupt EVs found in S1FR and S2FR. We found a disappearance of CD9^+^, CD63^+^, and CD41^+^ stained EVs through detergent treatment, validating the detected difference is not due to false-positive events derived from antibody aggregates (Supplementary Fig. S4).

In addition, we have also tested the effect of four different types of anticoagulant tubes (ACD, EDTA, heparin, and sodium citrate) in association with freeze/thaw cycle on differentially processed plasma (Supplementary Fig. S5). We observed the least change for 1000–3000 nm CD9^+^ and CD41^+^ EVs between S1 and S1FR in sodium citrate, while the lowest for 1000–3000 nm CD63^+^ EVs was from ACD. Overall, we observed a more than twofold increase for 150–1000 nm CD9^+^ and CD41^+^ EVs between S1 and S1FR in all anticoagulant tube types (Supplementary Fig. S5). In contrast, we observed no increase for 150–1000 nm CD63^+^ EVs between S1 and S1FR for any anticoagulant tube type. Throughout all anticoagulant tube types, minimal changes occurred between S2 and S2FR for CD9^+^, CD63^+^, and CD41^+^ EVs in both 150–1000 nm and 1000–3000 nm EV size ranges. In summary, our data indicated that freezing single spun plasma which contains residual platelets generated ex vivo EVs in a marker dependent manner, whereas no significant change was observed for residual platelet depleted plasma in the second spin prior to freezing.

### Ex vivo generated EVs are irreversible even after post-thaw processing

**Figure 5:**
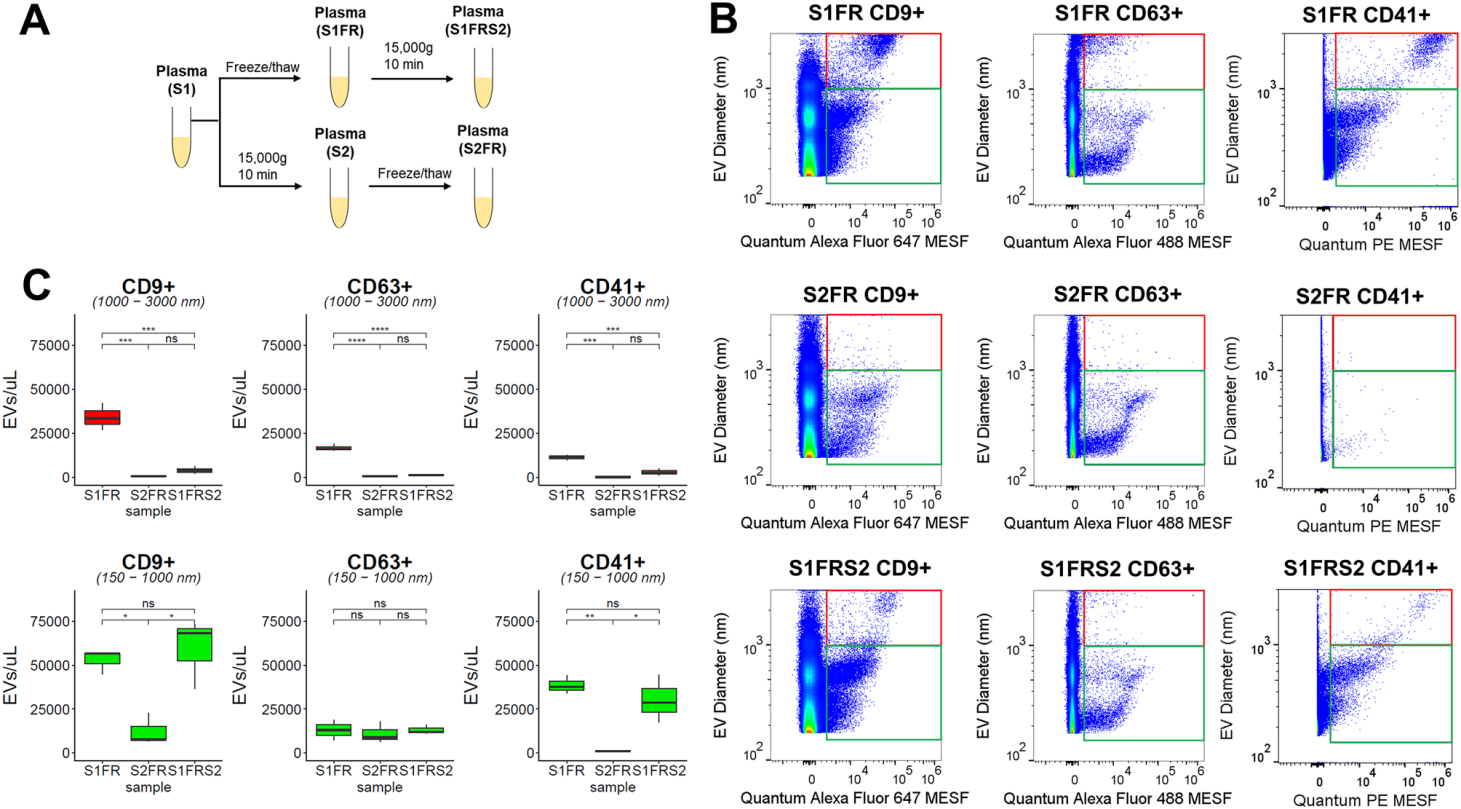
Effect of post-thaw processing on EVs using flow cytometry. (**A**) Schematic diagram of differentially processed plasma (S1, S2), respective freeze thaw samples (S1FR, S2FR), and secondary spin after post-freeze/thaw plasma S1FR (S1FRS2). (**B**) Representative flow cytometry dot plot of EV diameter (nm) versus fluorescent intensity in Quantum MESF units for CD9^+^ EVs, CD63^+^, and CD41^+^ EVs in S1FR, S2FR, and S1FRS2 conditions using FlowJo. Quantum Alexa Fluor 647 MESF is used for Alexa Fluor 647 conjugated CD9 stained plasma, Quantum Alexa Fluor 488 MESF is used for Alexa Fluor 488 conjugated CD63 stained plasma, and Quantum PE MESF is used for PE conjugated CD41 stained plasma. Events were gated from 150 to 1000 nm (green box) and from 1000 to 3000 nm (red box). (**C**) Box plot of CD9^+^, CD63^+^, CD41^+^ EV concentration from 1000–3000 nm (red) and 150–1000 nm (green) for S1FR, S2FR, and S1FRS2 using R. Statistical significance were obtained from three healthy volunteers for each freeze thaw processing condition using Tukey’s multiple comparisons (ns = not significant, *P* > 0.05; *P < 0.05, ***P < 0.001, ****P < 0.0001).

Next, to test if a post-thaw processing effectively removes ex vivo generated EVs, we performed centrifugation at 15,000 *g* for 10 min on S1FR plasma samples (S1FRS2) (Fig. 5A). We found S1FRS2 significantly depleted CD9^+^. CD63^+^, and CD41^+^ 1000–3000 nm populations associated with S1FR (Fig. 5B, C). The concentrations of 1000–3000 nm CD9^+^ EVs, CD63^+^ EVs, and CD41^+^ EVs in S1FR plasma sample were around 34,259/µl, 16,727/µl, and 11,279/µl respectively, while the concentrations of 150–1000 nm CD9^+^ EVs, CD63^+^ EVs, and CD41^+^ EVs in S1FR plasma sample were around 52,873/µl, 13,090/µl, and 38,588/µl respectively. When comparing between S1FR and S1FRS2, we observed 8.8-fold, 11.5-fold, and 3.9-fold decreases in 1000–3000 nm CD9^+^, CD63^+^, and CD41^+^ EVs respectively (Fig. 5C). Notably, these CD9^+^, CD63^+^, and CD41^+^ 1000–3000 nm levels in S1FRS2 were not significantly different compared to S2FR (S1FRS2 vs S2FR, ns) (Fig. 5C). Meanwhile, the levels of small and medium CD9^+^ and CD41^+^ EVs associated with S1FR remained significantly higher in post-thaw processed plasma S1FRS2 compared to S2FR (S1FRS2 vs S2FR, *P* < 0.05 for EV diameter 150–1000 nm) (Fig. 5B, C). On average, we observed 4.8-fold and 27.4-fold differences between S2FR and S1FRS2 in 150–1000 nm CD9^+^ and CD41^+^ EVs respectively. In contrast, we observed 150–1000 nm CD63^+^ EVs remained statistically unchanged (S1FRS2 vs S2FR, ns) (Fig. 5B, C). These CD9^+^, CD63^+^, and CD41^+^ 150–1000 nm levels in S1FRS2 were not significantly different compared to S1FR (S1FRS2 vs S1FR, ns). Collectively, our results revealed freezing residual platelets in S1 significantly generated small and medium CD9^+^ and CD41^+^ EVs ex vivo, which post-thaw processing could not remove. Meanwhile, we observed CD63^+^ EVs were retained regardless of spinning and post-thaw processing conditions.

### Distinct subsets of cf-mRNA influenced by differential centrifugation and post-thaw processing

**Figure 6:**
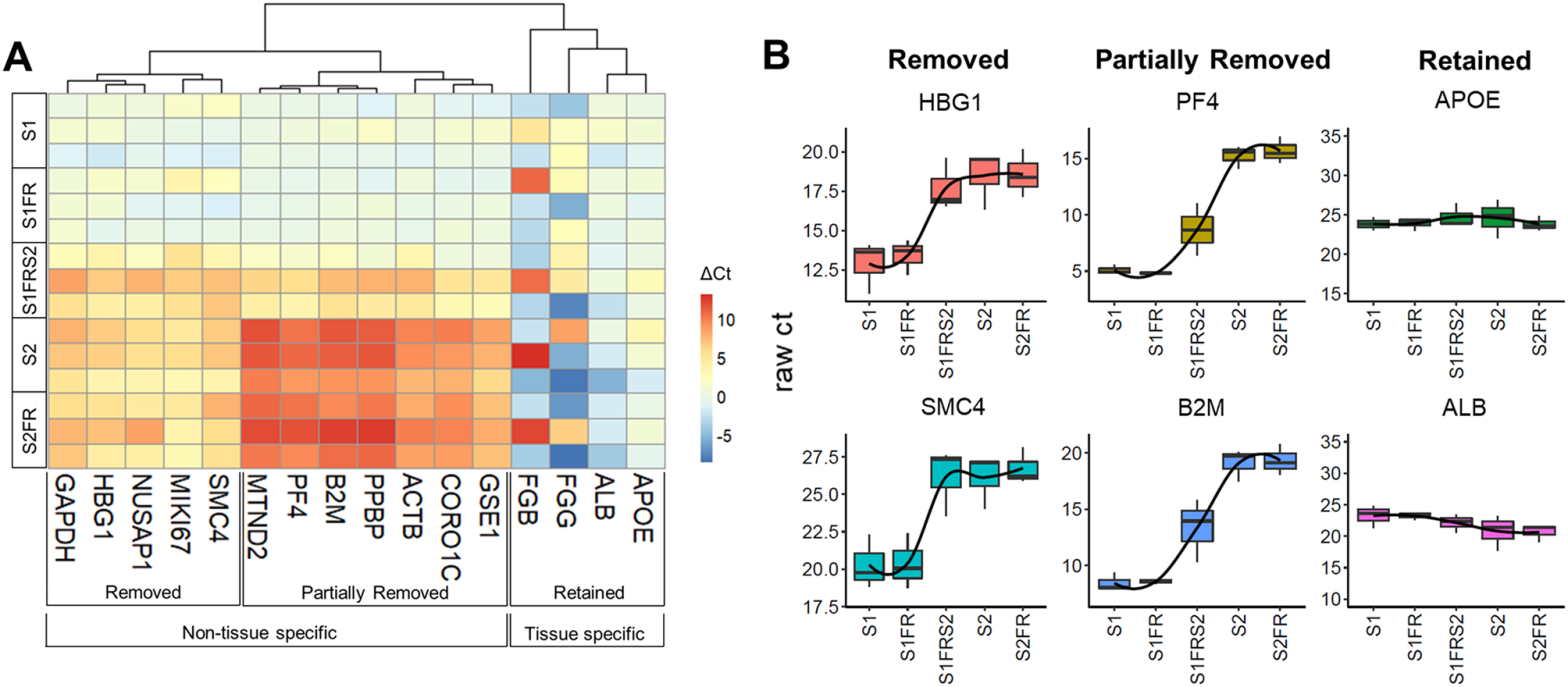
Effect of freeze thaw and post-thaw processing on cf-mRNAs using qRT-PCR. (**A**) Hierarchical clustering analysis of relative levels (in ΔCt) of 16 custom selected genes using RT-qPCR. Ct difference (ΔCt) between S1 and individual processing conditions are indicated from lowest (blue) to highest (red) using R. Non-tissue specific genes that are fully or partially removed, and tissue-specific genes which are retained in S1FRS2 with respect to S1 are shown. (**B**) Box plot of the median expression levels (in Cts) for representative non-tissue specific genes which are fully removed (i.e. HBG1 and SMC4) or partially removed (i.e. PF4, and B2M), and tissue-specific genes (i.e. APOE, ALB) which are retained in S1FRS2 with respect to S1 are shown using R. Higher raw Ct indicates lower levels of cfRNA transcripts.

Since platelets and EVs contain mRNA, we sought to determine if blood centrifugation and post-thaw processing affected cf-mRNA levels. We analyzed cf-mRNA profiles in single and second spin plasma freshly (S1 and S2), after freezing at − 80 °C (S1FR and S2FR), and for samples subjected to a second spin following S1FR processing (S1FRS2). We selected a panel of housekeeping, platelet and tissue-specific genes for multiplex RT-qPCR measurements (Fig. 6A, B). Hierarchical clustering analysis of relative gene expression between post-thaw processed samples revealed three distinct clusters (Fig. 6A). Overall, these clusters were either dependent (non-tissue specific) or independent (tissue specific) of post-thaw processing conditions, wherein non-tissue specific genes segregated into two clusters. The first cluster included genes (HBG1 and SMC4 for example), which could be removed by post-thaw processing and therefore were likely related to large EVs or platelets (Fig. 6A, B). The second cluster, including platelet genes and house-keeping genes such as PF4 and B2M, was partially removed by post-thaw processing and therefore was likely associated with ex vivo generated small and medium EVs which remained after post-thaw processing (Fig. 6A, B). Importantly, our results revealed that tissue specific gene signatures (such as genes expressed in liver tissue; including APOE and ALB) were retained regardless of spinning and post-thaw processing conditions (Fig. 6A, B), suggesting they are present in non-platelet small or medium EVs. The relationship of cf-mRNA transcripts with EV subpopulations requires further investigation and is the subject of future studies. Overall, as genes from different biological roles are uniquely affected by preanalytical differences, the selection of novel cfRNA biomarkers should consider the effects of preanalytical variability.

## Discussion

Circulating EVs and cf-RNA are promising biomarkers for disease diagnosis and prognosis^41–44^. However, significant variability in standardizing blood processing across published methods has led to a lack of reproducibility between studies^2,16,17,45^. In this study, we utilized multiparametric flow cytometry and cf-mRNA profiling to characterize preanalytical influences on EV and cf-mRNA subpopulations in plasma. We observed two distinct subpopulations by flow cytometry which are differentially impacted by centrifugation and post-thaw processing. Interestingly, we observed small and medium CD9^+^ EVs and CD41^+^ EVs were irreversibly generated via freezing single spun plasma while CD63^+^ EVs remained similar. Importantly, these ex vivo generated EVs could not be removed by additional centrifugation after freeze/thaw, and thereby can significantly affect downstream analyses. As a first in cf-mRNA studies, we also found groups of genes significantly, partially, or unaffected by post-thaw processing in plasma.

Since different types of EV purification methods (ultracentrifugation, density gradients, size-filtration, etc.) affect the yield and purity of EVs^46,47^, we chose to fluorescently label EVs directly in plasma using EV tetraspanin-specific antibodies with proper assay controls according to recent MIFlowCyt-EV reporting framework^48^. Previous study highlighted effects of centrifugation on pre-isolated EVs from platelets and erythrocytes by examining recovery of EVs through differential centrifugation^49^. However, their freeze–thaw cycle was performed on purified EVs, leading to no significant change across different temperatures of single freeze–thaw cycle. By examining EVs directly in plasma, we observed irreversible ex vivo generation of EVs and cf-mRNA subpopulations altered by differential centrifugation and post-thaw processing.

The choice of anticoagulant used during blood collection is another important preanalytical factor which influences downstream analyses and the extent of ex vivo EV release^15,50,51^. Other studies also have tested different anticoagulant tubes focusing on platelet microparticles or large EVs^28,50,52^. György et al. showed the highest CD42^+^/Annexin V^+^ platelet microparticle counts using flow cytometry were found in heparin compared to citrate and ACD tubes^50^. Lacroix et al., also examined Annexin V^+^ platelet microparticles using flow cytometry and found the most Annexin V^+^ platelet microparticles in decreasing order for EDTA, heparin, and citrate tubes^39^. After establishing light scattering calibration using flow cytometry, we have found the impact of anticoagulant tubes differed based on the markers being tested (CD9, CD63, or CD41) and size ranges (150–1000 nm EVs vs 1000–3000 nm EVs). Our study revealed that the combined effect of both freezing and anticoagulation with the highest fold change in 1000–3000 nm CD41^+^ EVs was found in heparin compared to EDTA, ACD, and sodium citrate tubes. However, EDTA generated the highest fold change in 1000–3000 nm CD9^+^ and CD63^+^ EVs. For 150–1000 nm EVs, EDTA generated the highest fold change for CD9^+^ and CD41^+^ EVs while the relative levels of CD63^+^ EVs were similar across all anticoagulants. Similar to Jayachandran et al. and Lacroix et al., we also found that counts of 1000–3000 nm large EVs were similar both before and after freezing double spun plasma^28,39^. Notably, while EDTA is commonly used for RNA analysis^53,54^, EDTA also caused the largest change in 150–1000 nm CD9^+^ and CD41^+^ EVs as well as 1000–3000 nm CD9^+^ and CD63^+^ EVs when freezing single spun plasma. Although we found the relative effect of anticoagulant types can be minimized by freezing samples as secondary spun plasma, it is important to test and choose anticoagulant types to fit specific research questions and downstream analysis.

Although previous studies highlighted the preanalytical influences on microparticle generation associated with platelet activations^40,49,55^, the effect of blood processing on EV subpopulations using flow cytometry with light scattering and fluorescence standardized calibration is lacking. The enumeration of microparticles in previous studies mostly utilized flow cytometry that was validated to discriminate between 0.5 and 0.9 μm Megamix beads^39,49^. Since considerable efforts have been directed to establish a standardized methodology for EV measurements by flow cytometry^30–34^, we applied this standardized approach to investigate enumeration of EVs influenced by preanalytical factors. Utilizing the FCMpass software developed by Welsh et al.^32–34^, we observed differential centrifugation results in distinct EV subpopulations within the diameter range between 150 and 3000 nm. In addition, our freeze thaw analysis further revealed ex vivo generated CD9^+^ EVs, adding to the body of literature on platelet-associated blood processing artefacts^2,16^. We addressed the importance of preanalytical influences on EVs using a standardized approach, which will improve the reproducibility with respect to effective EV diameter and given fluorochrome molecule standards across literatures.

Comprehensive assessment of light scattering sensitivity on multiple different flow cytometers was performed by Van der pol et al.^30^. For small particle detection, only a few flow cytometers detected more than three different sized reference beads using both side scatter (SSC) and forward scatter (FSC). Similarly, our instrument could not detect more than three sized reference beads by FSC. Instead, we utilized FCMpass software to calibrate SSC using the effective refractive index of EVs (Shell RI = 1.4800, Core RI: 1.3800, and shell thickness: 5 nm)^32^. Since the true refractive index of different EV subpopulations is currently unknown, the average EV refractive indices based on core–shell theory has been implemented^32,33^. Although EVs in the ~ 1000 nm diameter range may overlap with small platelets^56^, precise refractive indices which considers platelet granule content and shapes is currently unknown. Specific studies, which definitively parse EVs from small platelets and understanding refractive indices of EV subpopulations, are needed to better define EV physical characteristics and compositions.

How blood processing influences circulating microRNA has been previously shown^16,17^, and yet the impact on cf-mRNA is poorly understood. Cheng et al. provided preanalytical influences on miRNA expression due to differing residual platelet amount^17^. Conversely, our study investigated the impact of blood processing conditions through differential centrifugation, respective freezing condition, and post-thaw processing on cf-mRNA. We revealed cf-mRNA groups whose extent of preanalytical variability differed based on the degree of residual platelets in plasma. In particular, non-tissue specific genes were further classified as either partially or fully removed by post-freeze/thaw processing. Intriguingly, tissue-specific cf-mRNA were less prone to blood processing conditions, revealing them as potentially more robust biomarkers, or differentially associated with smaller vesicle subpopulations retained through centrifugation.

In conclusion, our study provides an assessment of the preanalytical effect of differential centrifugation and freeze/thaw cycles on plasma EVs and cf-mRNA. Employing multiparametric flow cytometry, our work provides insights into how preanalytical factors influence EV subpopulations and ex vivo release of EVs in association with residual platelets. Notably, these artifacts appear to be irreversible for CD9^+^ and CD41^+^ small and medium EVs and mRNA transcripts of genes present in platelets. However, an increasing number of other marker-specific studies, including non-platelet specific markers, will help to address the origin of the ex vivo EV generation observed in our study. Our results indicate distinct subpopulations of EVs and cf-mRNA are not removable by additional spinning after freeze/thaw. Therefore, consideration should be taken when analyzing EVs and cf-mRNA from banked plasma and designing robust EV and cf-mRNA based liquid biopsy tests.

## Supplementary Information

Supplementary Information.
