# Supplementary material for: Irreversible alteration of extracellular vesicle and cell-free messenger RNA profiles in human plasma associated with blood processing and storage
Source: Sci Rep. 2022 Feb 8;12:2099. doi: 10.1038/s41598-022-06088-9 (PMC8827089; doi:10.1038/s41598-022-06088-9)
Supplement: Supplementary file 1 — Supplementary Information. [file 41598_2022_6088_MOESM1_ESM.docx]

**Irreversible alteration of extracellular vesicle and cell-free messenger RNA profiles in human plasma associated with blood processing and storage.**

Hyun Ji Kim^1,2^, Matthew J. Rames^1,2^, Samuel Tassi Yunga^1,2^, Randall Armstrong^1^, Mayu Morita^4^, Anh T.P. Ngo^2^, Owen J.T. McCarty^2^, Fehmi Civitci^1^, Terry K. Morgan^1,2,4^, and Thuy T. M. Ngo^1,2,3,*^

1. *Cancer Early Detection Advanced Research Center, Knight Cancer Institute (CEDAR), Oregon Health and Science University, Portland, OR, USA*
2. *Department of Biomedical Engineering, Oregon Health and Science University, Portland, OR, USA*
3. *Department of Molecular and Medical Genetics, Oregon Health and Science University*
4. *Department of Pathology, Oregon Health and Science University*

*Correspondence to:

Thuy T. M. Ngo

Cancer Early Detection Advanced Research Center (CEDAR), Knight Cancer Institute

2720 SW Moody Ave, KR-CEDR, Portland, OR 97201

Oregon Health & Science University

[ngth@ohsu.edu](mailto:ngth@ohsu.edu)

**Supplementary Figures**

| 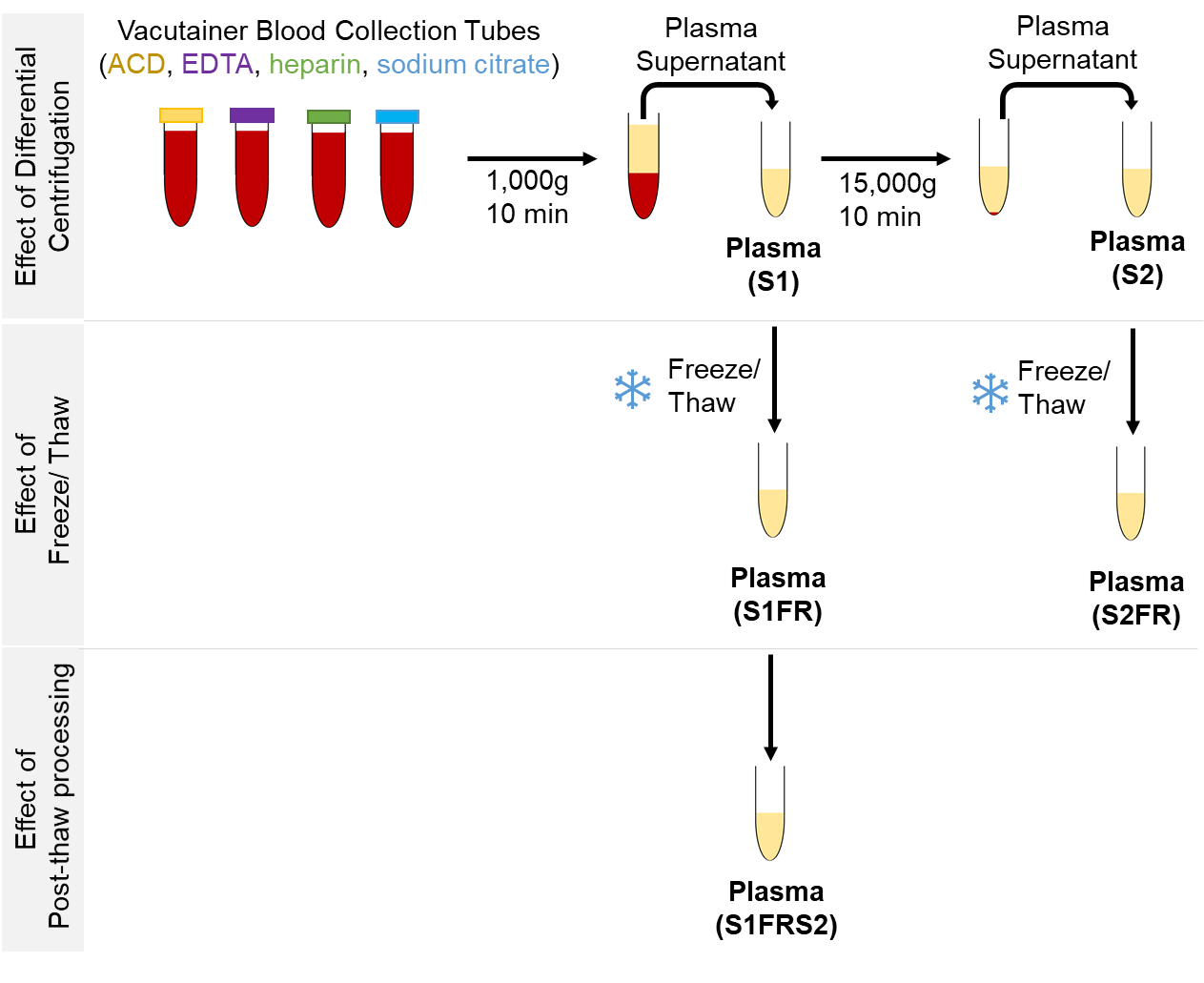 |
| --- |
| **Supplementary Figure S1 \| Schematic diagram of overall blood processing workflow**  Whole blood samples were collected into four different types of vacutainer blood collection tubes ACD, EDTA, heparin, and sodium citrate in the study. Effect of differential centrifugation was evaluated between single spun plasma at 1,000 × g for 10 minutes versus additional spin at 15,000 × g for 10 minutes (S1 vs S2). Effect of freeze/thaw was evaluated comparing freeze thaw cycle on S1 or S2 (S1FR vs S2FR). Effect of post-thaw processing was evaluated comparing an additional spin at 15,000 × g for 10 minutes performed on S1FR (S1FRS2). |

| 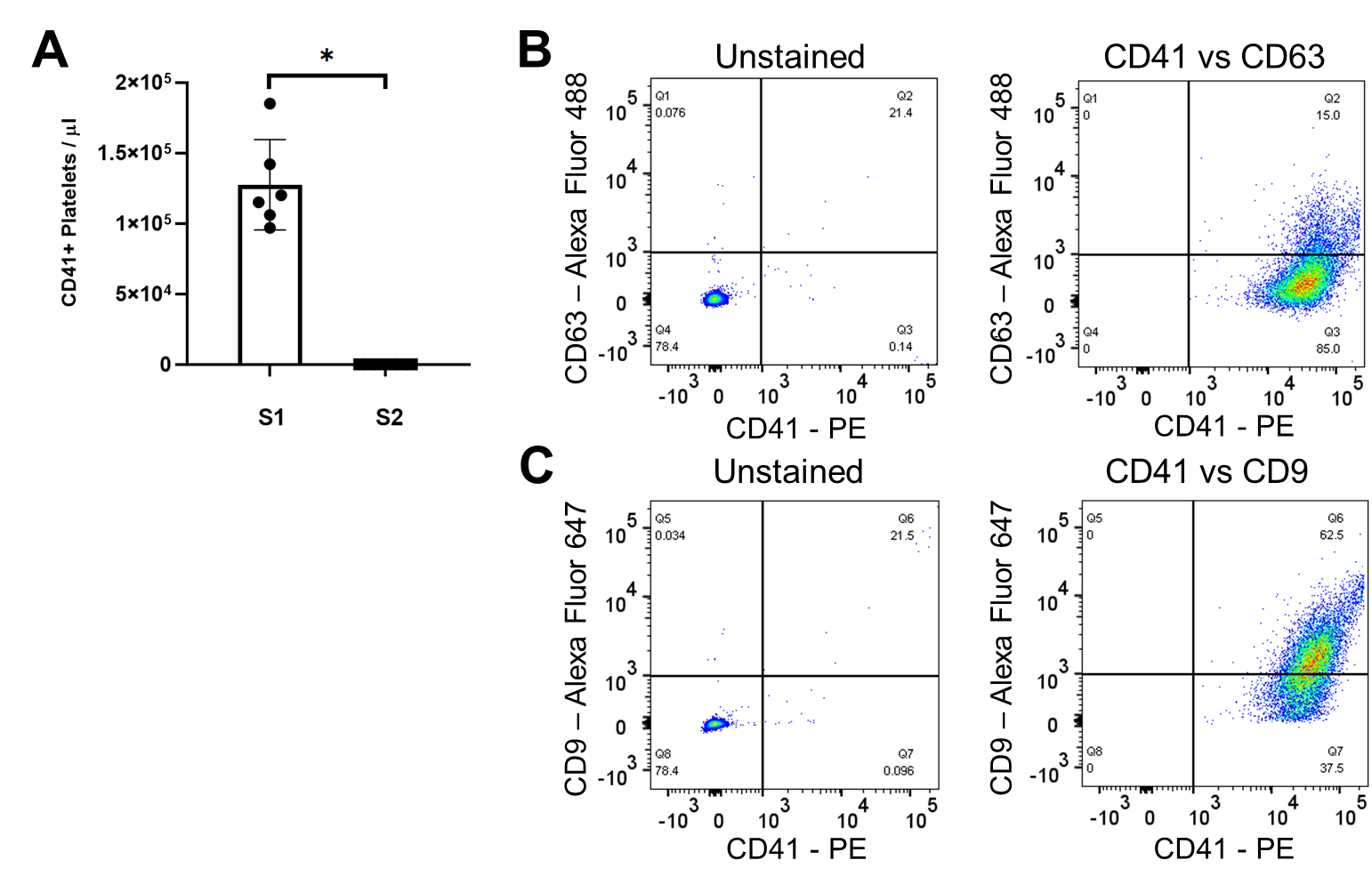 |
| --- |
| **Supplementary Figure S2 \| Characterization of platelets using flow cytometry**  (A) Platelet concentration in differentially processed plasma from three healthy individuals (n=3) was measured in independent technical replicates using flow cytometry. The error bar represented standard deviations for the indicated blood processing conditions. P-value was calculated using Wilcoxon test (*P <0.05). (B) Representative flow cytometry dot plot of PE vs. Alexa Fluor 488 on unstained platelets and CD41^+^ vs. CD63^+^ platelets using FlowJo. (C) Representative flow cytometry dot plot of PE vs. Alexa Fluor 647 on unstained platelets and CD41^+^ vs. CD9^+^ platelets using FlowJo. |

| 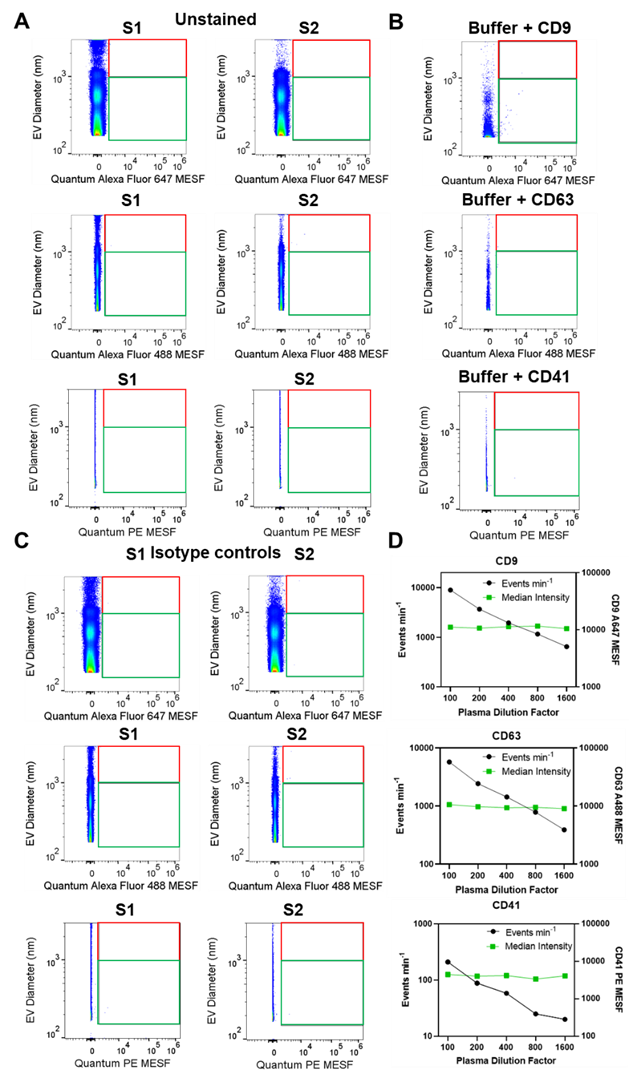 |
| --- |
| **Supplementary Figure S3 \| Flow cytometry experimental assay controls**  Representative flow cytometry dot plot of antibody with (A) unstained S1 and S2 plasma, (B) Alexa Fluor 647 conjugated CD9, Alexa Fluor 488 conjugated CD63, or PE conjugated CD41 with buffer alone, and (C) Alexa Fluor 647 conjugated, Alexa Fluor 488 conjugated, or PE conjugated isotypes in S1 and S2 plasma using FlowJo. (D) Scatter plots of CD9^+^, CD63^+^, and CD41^+^ EVs dilution controls. |
| 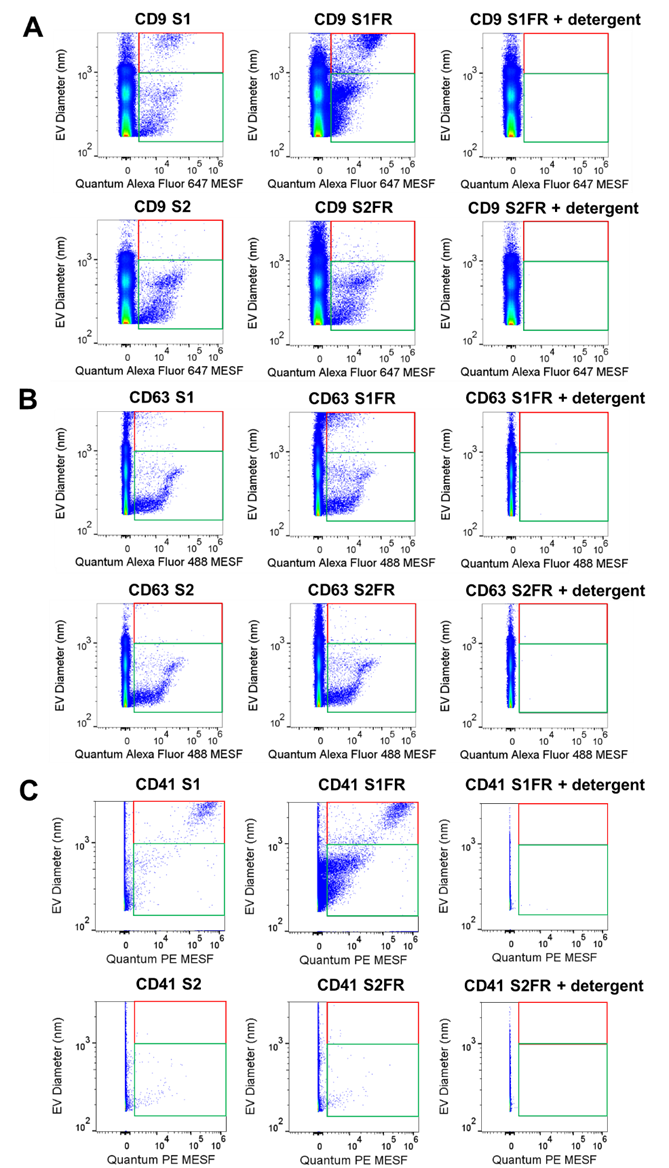 |
| **Supplementary Figure S4 \| Freeze-thaw effect and detergent treatment of plasma EVs**  Representative dot plots EVs from S1 and S2 plasma, respective freeze-thaw processing (S1FR and S2FR), and detergent controls on (A) CD9^+^ EVs, (B) CD63^+^ EVs, and (C) CD41^+^ EVs using FlowJo. S1FR and S2FR were treated with detergent (2% SDS) prior to staining. |
| 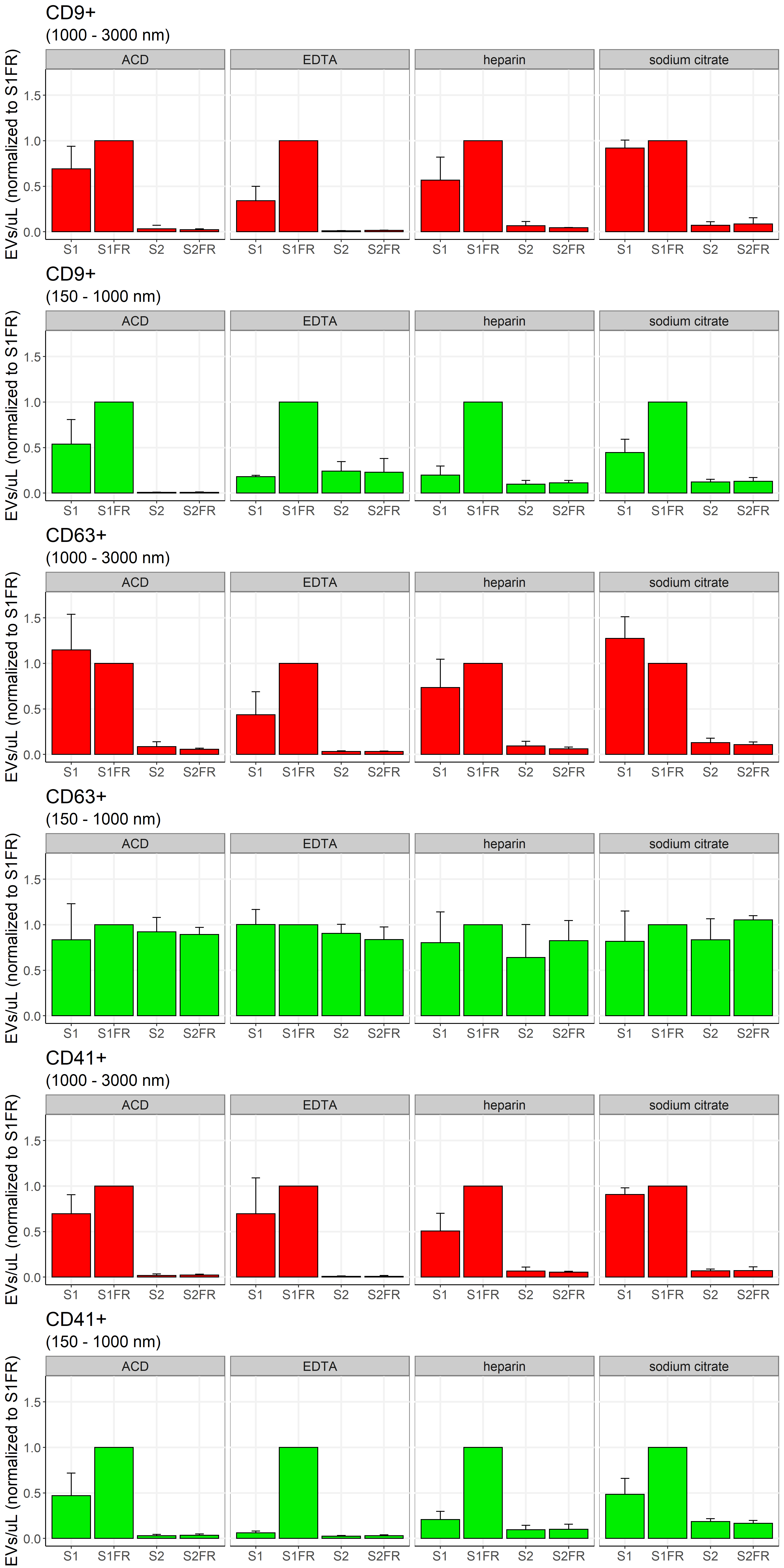 |
| **Supplementary Figure S5 \| Effect of Anticoagulants through differential centrifugation and freeze-thaw cycles on EVs using flow cytometry**  Representative bar graphs of CD9^+^, CD63^+^, and CD41^+^ normalized EV concentration from 1,000 – 3,000 nm (red) and 150 – 1,000 nm (green) measured in differentially processed plasma (S1, S2) and respective freeze-thaw processes (S1FR, S2FR) from three healthy individuals (n=3) using four different anticoagulant tubes (ACD, EDTA, heparin, and sodium citrate). EV concentration values were normalized relative to S1FR for each individual using R. The error bar represented the standard deviation for each indicated blood processing condition. |
